# Efficient and Sustainable Platform for Preparation of a High-Quality Immunoglobulin G as an Urgent Treatment Option During Emerging Virus Outbreaks

**DOI:** 10.3389/fimmu.2022.889736

**Published:** 2022-05-17

**Authors:** Tihana Kurtović, Sanda Ravlić, Adela Štimac, Sanja Mateljak Lukačević, Ana Hećimović, Saša Kazazić, Beata Halassy

**Affiliations:** ^1^ Centre for Research and Knowledge Transfer in Biotechnology, University of Zagreb, Zagreb, Croatia; ^2^ Center of Excellence for Virus Immunology and Vaccines, Zagreb, Croatia; ^3^ Croatian Institute of Transfusion Medicine, Zagreb, Croatia; ^4^ Division of Physical Chemistry, Ruđer Bošković Institute, Zagreb, Croatia

**Keywords:** convalescent plasma processing, polyclonal anti-COVID-19 IgG, IgG subclass, passive immunotherapy, wild-type SARS-CoV-2 neutralization assay, caprylic acid precipitation, ion-exchange chromatography, mass spectrometry

## Abstract

During the pre-vaccine era of the COVID-19 pandemic convalescent plasma has once again emerged as a major potential therapeutic form of passive immunization that in specific cases still represents irreplaceable treatment option. There is a growing concern that variable concentration of neutralizing antibodies, present in convalescent plasma which originates from different donors, apparently affects its effectiveness. The drawback can be overcome through the downstream process of immunoglobulin fraction purification into a standardized product of improved safety and efficacy. All modern procedures are quite lengthy processes. They are also based on fractionation of large plasma quantities whose collection is not attainable during an epidemic. When outbreaks of infectious diseases are occurring more frequently, there is a great need for a more sustainable production approach that would be goal-oriented towards assuring easily and readily available immunoglobulin of therapeutic relevance. We propose a refinement strategy for the IgG preparation achieved through simplification and reduction of the processing steps. It was designed as a small but scalable process to offer an immediately available treatment option that would simultaneously be harmonized with an increased availability of convalescent plasma over the viral outbreak time-course. Concerning the ongoing pandemic status of the COVID-19, the proof of concept was demonstrated on anti-SARS-CoV-2 convalescent plasma but is likely applicable to any other type depending on the current needs. It was guided by the idea of persistent keeping of IgG molecules in the solution, so that protection of their native structure could be assured. Our manufacturing procedure provided a high-quality IgG product of above the average recovery whose composition profile was analyzed by mass spectrometry as quality control check. It was proved free from IgA and IgM as mediators of adverse transfusion reactions, as well as of any other residual impurities, since only IgG fragments were identified. The proportion of S protein-specific IgGs remained unchanged relative to the convalescent plasma. Undisturbed IgG subclass composition was accomplished as well. However, the fractionation principle affected the final product’s capacity to neutralize wild-type SARS-CoV-2 infectivity, reducing it by half. Decrease in neutralization potency significantly correlated with the amount of IgM in the starting material.

## 1 Introduction

In the early stages of the COVID-19 pandemic no proven vaccine or effective drug was immediately available. Convalescent plasma, an old principle used with apparent success in many prior outbreaks, has once again emerged as a major potential therapeutic form of passive immunization (1–3). If the amount of specific antibodies is sufficient to alter the outcome of a disease, its administration early in the course of hospitalization is associated with viral load reduction, clinical improvement and decrement in mortality (4–9). Unlike other therapies, convalescent plasma becomes available as soon there are survivors, without any need for further development (1). Its beneficial effect is attributed to the pathogen-specific neutralizing antibodies as the main driver of improved outcome whose action might be influenced by numerous other co-factors, some of which may exhibit detrimental impact (10). Further, there is a growing concern that variation in the concentration of neutralizing antibodies present in convalescent plasma and the subsequent lack of standardized doses between patients apparently affect its effectiveness (11). And last but not least, when applied to the patient, convalescent plasma becomes diluted to the concentration that might be inadequate for a successful treatment outcome. Many of the recognized drawbacks can be overcome through the downstream process of purification, concentration and formulation of immunoglobulin fraction into a standardized product of improved safety and efficacy that at the same time enables removal of aggregates and other contaminants associated with adverse reactions, as well as reduces the risk of the transmission of blood-borne pathogens (12).

From the first half of the 20th century many suitable methods to separate immunoglobulin G (IgG) from human plasma at the industrial scale have been developed (13–15). Current core fractionation technology largely relies on a well-established backbone process encompassing cryoprecipitation and cold-ethanol precipitation (16, 17). Over the years its complexity has remarkably increased due to the implementation of additional steps, not only to improve product’s purity, enhance its recovery and assure viral inactivation or removal, but also to isolate new clinically useful plasma proteins from existing fractions. The soundness and large-scale adaptability of the technology, on one hand, and the rigidity of the current regulatory framework, on the other, explains why it still remains the main method in the field of modern plasma fractionation.

IgG is generally regarded as a safe therapy. However, if treatment involves high doses administered by *i.v.* route, severe adverse events due to some plasma protein contaminants may occur. Those associated with immunoglobulin isotypes other than IgG are of special concern because of their clinical significance (18). IgA can cause selective IgA deficiency-mediated anaphylactic transfusion reaction (19). It is a life-threatening complication that occurs within one hour of transfusion of blood products in recipients who are IgA-deficient and have anti-IgA antibodies. IgM is a trigger of hemolytic transfusion reaction (20). When intravenous immunoglobulin contains anti-A or anti-B antibodies, in transfused A, B and AB patients they act as isoagglutinins, recognizing respective blood group antigens. Their binding activates terminal complement components, which leads to intravascular hemolysis due to formation of membrane-attack complex, destruction of red-cell membranes and releasement of free hemoglobin into the intravascular space. End-organ damage, including acute tubular necrosis and renal failure, eventually may ensue. In-view-of transfusion reaction severity, design of innovative purification approaches that will be committed towards removal of IgA and IgM is demanded for ensuring highest safety standards of IgG-based therapeutics.

When epidemics of infectious diseases are occurring more frequently and spreading faster and further than ever, there is a great need for a more sustainable production approach that would be goal-oriented towards assuring easily and readily available plasma-derived immunoglobulin of therapeutic relevance. Here, we propose a refinement strategy for the preparation of a ready-to-use IgG, achieved through simplification and reduction of number of processing steps. It has been demonstrated on anti-SARS-CoV-2 convalescent plasma and consisted of caprylic acid-mediated fractionation, depletion of precipitating agent from the IgG-containing fraction and its final polishing by the flow-through chromatography. Completely pure IgG of above the average yield, preserved neutralization potency and undisturbed subclass composition was obtained. The recovery of active drug was precisely quantified in every processing step, enabling accurate estimation of the procedure’s cost-effectiveness.

## 2 Materials and Methods

### 2.1 Ethics Statements

The study involving human participants was reviewed and approved by Croatian Ministry of Health (023-03/20-01/235; permission No. 534-04-3-2/2-20-11). The approval was based on the positive opinion of the Ethical Committee of Croatian Institute of Transfusion Medicine (003-06/20-04/02, opinion No.251-541-06/6-20-2). All COVID-19 convalescent plasma (CCP) donors were informed about the study and gave written informed consent.

### 2.2 COVID-19 Convalescent Plasma

CCP was collected by apheresis procedure from recovered and healthy patients who had been asymptomatic for ≥ 28 days. All consenting donors had a documented history of laboratory-confirmed SARS-CoV-2 infection based on RT-PCR test and were eligible for donations according to the standard blood donor criteria. Plasma from each individual was screened for the presence of anti-SARS-CoV-2 neutralizing antibodies, that were quantified by ED_50_ assay. Three donation samples proven positive were randomly chosen as the starting material for downstream processing which was independently performed eight times in total.

### 2.3 SARS-CoV-2 Working Stock

SARS-CoV-2 working stock for neutralization assay was prepared, characterized and stored as already described (21, 22). The virus for ELISA was purified from Vero E6 cell culture supernatant that was collected 2 days post-infection, heat-inactivated at 65°C for 30 min (23) and centrifuged at 141,000 × *g* for 4 h. The pellet resuspended in PBS was used as coating antigen.

### 2.4 Reagents

Bovine serum albumin (BSA), caprylic acid (≥ 98%), dithiothreitol (DTT), iodoacetamide (IAA), HPLC grade trifuoroacetic acid (TFA), 2-(N-morpholino) ethanesulphonic acid (MES) monohydrate, *o*-phenylenediamine dihydrochloride (OPD), thimerosal, Tris base, sodium dodecyl sulphate (SDS), ethylenediaminetetraacetic acid (EDTA), Tween 20, thyroglobulin, γ-globulin, ovalbumin and ribonuclease A were from Sigma-Aldrich (USA). Sodium acetate was from Fluka (Germany). HPLC grade acetonitrile was purchased from Merck (Germany). All other chemicals for buffers and solutions were from Merck (Germany), unless otherwise stated.

### 2.5 Plasma Downstream Processing

#### 2.5.1 Caprylic Acid Precipitation

In preliminary experiment CCP, aliquoted and stored at -20 °C until use, was thawed at room temperature and incubated at 56 °C for 1 h in a thermomixer (Eppendorf, Germany). After centrifugation at 3,200 × *g* for 45 min and discarding of the pellet, caprylic acid was added in a dropwise manner so that final concentrations of 1-7% (*V/V*) in 2-fold diluted reaction mixtures (*V =* 1 mL) were prepared, each in duplicate. Saline was used as diluent. Precipitation was performed by vigorous stirring (850 rpm) at 23 °C for 1 h, followed by centrifugation (3,200 × *g*, 45 min). Minimal caprylic acid concentration giving the highest IgG purity of acceptable yield was chosen as the most beneficial, as described in Results. In all subsequent experiments the precipitation step was performed under optimized conditions. Furthermore, the whole procedure was scaled up 40-fold. IgG-containing supernatant was first filtered through a hydrophilic cloth and a cellulose acetate filter with a pore size of 5 μm (Sartorius, Germany), and afterwards diafiltrated into 20 mM sodium acetate buffer, pH 5.0 using a Vivacell device (Sartorius, Germany) with a 100 kDa molecular weight cut-off (MWCO) polyethersulfone membrane. Several repeated cycles of concentration and dilution were performed to achieve approximately 4 logs of matrix exchange.

#### 2.5.2 Flow through HPLC

Diafiltrated IgG sample was loaded (2 mL per run) to the pre-equilibrated CIM QA disk (*V* = 0.34 mL) (BIA Separations, Slovenia) with 20 mM sodium acetate buffer, pH 5.0 at a flow rate of 1 mL min^-1^ on an ÄKTA chromatography system (GE Healthcare, USA). The absorbance was monitored at 280 nm. After collecting the flow-through fraction, the bound components were eluted from the column with binding buffer containing 1 M NaCl. During elution step flow rate was 2 mL min^-1^. Flow-through fractions from all runs were pooled into the final product and concentrated on a membrane with a MWCO of 100 kDa to achieve IgG concentration in the range of that measured in CCP.

### 2.6 Assessment Methods

#### 2.6.1 SDS-PAGE, 2D Gel Electrophoresis and In-Gel Digestion

Purity in each processing step was examined by SDS-PAGE on NuPAGE 4-12% Bis-Tris gel with MES as running buffer under non-reducing conditions in an Xcell SureLock Mini-Cell, according to the manufacturer’s procedure (Invitrogen, USA). Electrophoresis was performed at 200 V for 45 min. Staining was carried out with acidic Coomassie Brilliant Blue (CBB) R250 solution. For densitometric analysis CBB R250-stained image was recorded in trans-illumination mode with Amersham Imager 680 (GE Healthcare, USA) and processed by background subtraction using the rolling ball (*r* = 20 mm) algorithm, followed by analysis of the relative band volume within a lane.

Isoelectric focusing, the first dimension of 2D gel electrophoresis, was performed in a ZOOM IPGRunner Mini-Cell on an immobilized pH gradient (IPG) strip (7 cm long, linear pH 3-10) (Invitrogen, USA) that was previously rehydrated with the final product (*m* = 250 μg). Details are given in the manufacturer’s protocol. The following step voltage protocol was applied: 200 V for 20 min, 450 V for 15 min, 750 V for 15 min and 2,000 V for 5 h. For the second dimension, the IPG strip was reduced with 20 mM DTT, alkylated with 125 mM IAA and inserted into the well of a NuPAGE 4-12% Bis-Tris ZOOM gel (Invitrogen, USA). Electrophoresis was performed under conditions described above. Excised protein spots obtained by 2D gel electrophoresis of IgG sample were in-gel digested, as described (24).

#### 2.6.2 LC-MS/MS Analysis

The digested samples were reconstituted in water : acetonitrile (ACN) = 50 : 50 (*V*/*V*) containing 0.1% formic acid (FA) (*V*/*V*). For each LC-MS/MS run, 5 µL of the sample was injected in the sample loop of the Thermo Fischer UltiMate 3000 RSLCnano system. All peptides in mixtures were first desalted by running HPLC solvent A (2% ACN (*V*/*V*) in 0.1% FA (*V*/*V*)) at 40 µL min^-1^ microflow rate across the trap column (Acclaim PepMap100 C18, 5 µm, 100 Å, 1 mm i.d. × 15 mm) for 1 minute. Peptides were separated on a capillary analytical column (Acclaim PepMap RSLC C18, 2 µm, 100 Å, 300 µm i.d. × 15 cm) during a 45-min long segmented gradient run of 5-90% HPLC solvent B [98% ACN (*V*/*V*) in 0.1% FA (*V*/*V*)] at a flow rate of 3.5 µL min^-1^ and eluted into a Bruker Daltonik amaZon ETD Ion Trap mass spectrometer for MS and MS/MS mass measurement. MS data acquisition was conducted in the positive ion mode. The mass spectrometer was operated in the data-dependent mode to switch between MS and MS/MS acquisition automatically. The MS parameters used for the data acquisition were as follows − MS range: 300-1500 *m*/*z*; MS/MS range: 100-2400 *m*/*z*; capillary voltage: 4500 V; dry gas temperature: 200 °C; dry gas flow: 5 L min^-1^; collision gas: helium; fragmentation voltage: 1 V; enhanced resolution scan mode: 8100 *m*/*z* per second. A maximum of three precursors were selected for the fragmentation. The charge states for tryptic digested samples were selected as 2, 3, > 3 and isolation width was 4.0 *m*/*z* units.

The mgf files were generated from the raw mass spectrometric data using DataAnalysis software (version 4.0 SP4, Bruker Daltonik). Protein identification was proceeded on Mascot Server (version 2.3.02, Matrix Science) through BioTools (version 3.2, Bruker Daltonik) interface with the following parameters − database: UniProtKB/Swiss-Prot (release 01/2022, taxonomy “all entries”); peptide tolerance: 20 ppm, MS/MS tolerance: 0.5 Da; amino acid modifications: cysteine (carbamidomethylation) as a fixed modification and methionine (oxidation) as variable modification; the number of missed cleavages: 2; enzyme specificity (selected charge states): trypsin (2+,3+, 4+); the database searches: decoy enabled.

#### 2.6.3 Total Protein Concentration

Throughout the isolation procedure total protein concentration was estimated spectrophotometrically according to the Eq (1) (25),


(1) $$\gamma \left[{\text{mg mL}}^{-1}\right]\;=\;\left(A_{228.5\text{ nm}}–A_{234.5\text{ nm}}\right)\;\times f\times \text{dilution factor}$$

where Ehresmann’s factor “*f*” for human IgG of 0.2597 was used. It was calculated from the *A*_228.5 nm_ and *A*_234.5 nm_ values obtained for intravenous immunoglobulin (IVIG) preparation (Institute of Immunology Inc., Croatia) and its nominal concentration, precisely measured by the Kjeldahl method for quality control purposes. Appropriate dilution of each sample was independently prepared minimum three times to obtain the mean value for further calculation of yield and purity.

#### 2.6.4 Size-Exclusion Chromatography

Size-exclusion chromatography (SEC), which was employed to monitor IgG purity throughout the production process, was performed on TSK-Gel G3000SWXL column (7.8 × 300 mm) (Tosoh, Japan) with 0.1 M phosphate-sulphate running buffer, pH 6.6 at a flow rate of 1 mL min^-1^ on a Shimadzu HPLC system (Shimadzu, Japan). The samples (5 mg mL^-1^) were loaded to the column in a volume of 50 μL per run. The effluent was monitored at 280 nm. For determination of the IgG molecular weight, thyroglobulin (*M*_r_ 665,000), γ-globulin (*M*_r_ 150,000), ovalbumin (*M*_r_ 44,300) and ribonuclease A (*M*_r_ 13,700) were used as standards. Correction factor corresponding to deviation of molecular mass of analyzed IgG, determined according to the calibration curve, from its theoretical molecular mass, was included in the calculation.

#### 2.6.5 Reverse Phase HPLC for Estimation of Residual Caprylic Acid

Quantification of residual caprylic acid was determined by the validated HPLC method (26). Briefly, HPLC analysis was performed on RP C18 column (2.1 × 150 mm) (Shimadzu, Japan) with 0.05% TFA in water : ACN = 50 : 50 (*V*/*V*) as a mobile phase in isocratic mode at a flow rate of 0.2 mL min^-1^ on a Shimadzu HPLC system (Shimadzu, Japan). Wavelength of 210 nm was used for detection. The injection volume was 5 µL and the total run time was 10 min. Before analysis, interfering immunoglobulins were precipitated with one volume of ACN. The amount of caprylic acid was determined in samples before and after diafiltration and in every filtrate. The samples with low concentration of caprylic acid (below limit of quantification) were spiked with a known amount before HPLC analysis.

#### 2.6.6 ELISA for Determination of IgG, IgM and IgA Concentration

Determination of immunoglobulins in every CCP and the samples obtained by its processing was performed by coating the microtiter plate with 100 µL/well of the primary antibody solution (1 μg mL^-1^) in 50 mM carbonate buffer, pH 9.6, and left overnight at room temperature (RT). For IgG, IgA and IgM quantification anti-human IgG (Fc specific), anti-human IgA (α-chain specific) or anti-human IgM (μ-chain specific), respectively, all produced in goat (Sigma-Aldrich, USA), were used as capture antibodies. After washing and blocking with 2% (*w*/*V*) BSA in PBS with 0.05% (*V*/*V*) Tween 20 (250 µL/well) for at least 2 h at 37 °C, samples were added in 2-fold serial dilutions in duplicates (100 µL/well) and left overnight at RT. For IgG determination completely pure IgG-based final product, which has been precisely quantified, served as standard for analysis of the matching CCP and all intermediates in its downstream processing (27). IgA and IgM contents were determined using pure human IgA or IgM (Sigma-Aldrich, USA) as standards. In the next step, washed plates were incubated with 100 µL/well of anti-human IgG (Fab specific)-peroxidase antibody (20,000-fold diluted), anti-human IgA (α-chain specific)-peroxidase antibody (10,000-fold diluted) or anti-human IgM (μ-chain specific)-peroxidase antibody (10,000-fold diluted), respectively, all produced in goat (Sigma-Aldrich, USA), at 37 °C for 2 h. Following washing, addition of OPD in citrate-phosphate buffer, pH 5.0 (0.6 mg mL^-1^, 100 µL/well) and 30 min-long incubation in the dark at RT, the enzymatic reaction was stopped with 1 M H_2_SO_4_ (50 µL/well) and the absorbance at 492 nm was measured. IgG, IgA or IgM concentrations from at least three independently performed assays were used for calculation of the respective immunoglobulin class recovery, using Eq (2),


(2) [(γ(Ig)× dilution factor)/γ(Ig) in starting material]×100%

#### 2.6.7 SARS-CoV-2-Specific ELISA for Assessment of IgG Subclass Distribution

The assay was performed following the general principles described in Section 2.6.6. The plate was coated with inactivated and concentrated SARS-CoV-2 virus. After blocking, CCP and the final product of its refinement, adjusted to the same total IgG concentration, were added, together with the plasma from SARS-CoV-2 negative donor. Each sample was analyzed in hexaplicate. For detection of the respective IgG subclass, monoclonal anti-human IgG1 (1,500-fold diluted), anti-human IgG2 (1,000-fold diluted), anti-human IgG3 (5,000-fold diluted) or anti-human IgG4 antibody (2,500-fold diluted), all produced in mouse (Sigma-Aldrich, USA), were used. They were detected by anti-mouse IgG (whole molecule) from goat conjugated with peroxidase (5,000-fold diluted) (Cappel, USA). Ratio of *A*_492 nm_ values obtained for the final product and CCP was used as a measure of shift in the IgG subclass distribution.

#### 2.6.8 S Protein-Specific ELISA for Evaluation of IgG Recovery

IgG fraction against SARS-CoV-2 spike (S) protein was measured by commercial Anti-SARS-CoV-2 ELISA assay (Euroimmun Medizinische Labordiagnostika AG, Germany) that uses S1 domain recombinantly produced in HEK 293 cells as a coating antigen, according to the provided instructions. Ratio of *A*_450 nm_ values obtained for the final product and CCP, analyzed in the same total IgG concentration in duplicates, was used as a measure of shift in the distribution of S protein-specific IgGs.

#### 2.6.9 Wild-Type SARS-CoV-2 ED_50_ Assay

ED_50_ assay was performed in 96-well tissue culture microplates (TPP, Switzerland), as described (22). Two-fold serial dilutions of every sample, each in octaplicate (50 μL/well), were preincubated with approximately 20 CCID_50_ of the wild-type SARS-CoV-2 working stock (50 μL/well) at 37 °C and 5% CO_2_ for 90 minutes. In the next step Vero E6 cell suspension was added (3 × 10^5^ mL^-1^, 100 µL/well). The plates were incubated at 37 °C and 5% CO_2_. On the fourth day wells with cytopathic effect (CPE) were counted. The effective dose 50 (ED_50_), the amount of undiluted sample that inhibits CPE in 50% of the infected wells, was calculated according to Spearman-Kärber method. Neutralization potency was expressed as number of ED_50_ doses in 1 mL. Anti-SARS-CoV-2 in-house standard was included in every experiment, representing internal reference. Factor of its deviation from the nominal value was used for correction of results. In-house standard was calibrated to the 1^st^ WHO International Standard for anti-SARS-CoV-2 (NIBSC, UK), enabling expression of neutralization potency in IU mL^-1^ (22). Samples were independently analyzed at least three times.

#### 2.6.10 Data Analysis

The results of each analysis are expressed as the average (arithmetic or geometric mean) of *n* measurements ± confidence interval (CI), unless stated otherwise. Relationship between the degree of discrepancy in specific activity of the final product with respect to the CCP and the IgM share in immunoglobulin pool of the starting material was assessed by Pearson’s correlation coefficient (*r*), calculated using IBM SPSS Statistics for Macintosh, Version 28.0 (IMB Corp., USA).

## 3 Results

### 3.1 Refinement Protocol

**Figure 1:**
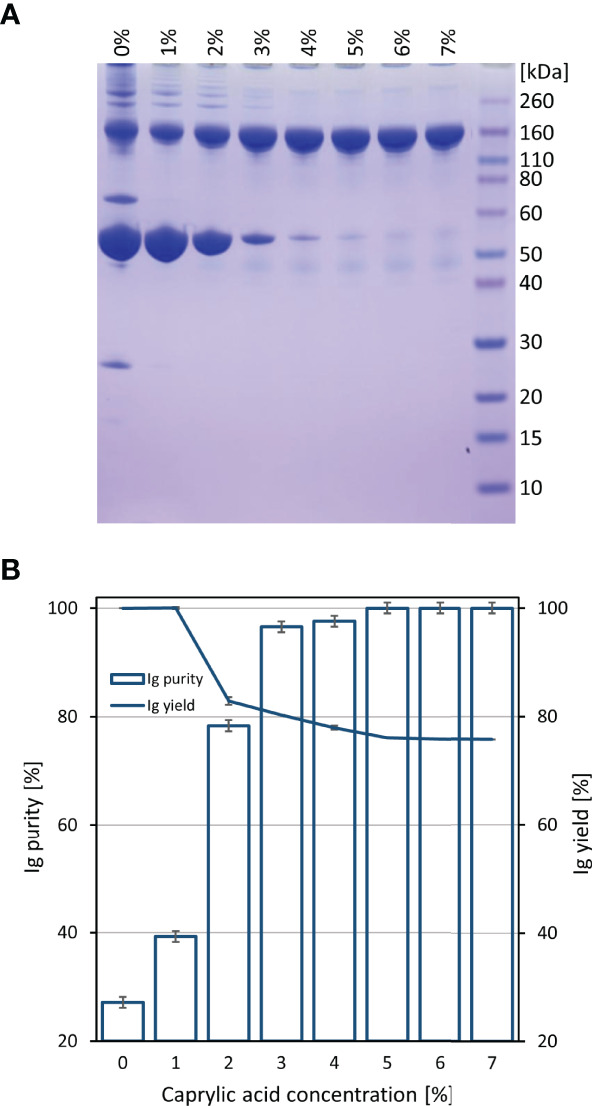
Preliminary determination of optimal caprylic acid concentration for precipitation step of the purification protocol. SDS-PAGE **(A)** and densitometric analysis **(B)** of samples obtained by 0-7% (*V*/*V*) caprylic acid. Results (IgG purity and yield) are given as mean +/- 95% CI (*n* = 2).

Initially, COVID-19 convalescent plasma (CCP) was heat-treated and fractionated by caprylic acid precipitation for removal of non-immunoglobulin proteins. Samples were analyzed by SDS-PAGE (**Figure 1A**). According to densitometric analysis (**Figure 1B**), all investigated concentrations in the range of 1-7% (*V*/*V*) in two-fold diluted reaction mixtures, except the lowest one, affected recovery of immunoglobulins in supernatant, causing approximately 20% of their loss, which appeared gradually more pronounced by the use of higher caprylic acid quantities. Since the 5% final volume ratio was identified as the minimal amount of precipitating agent that produced highly pure immunoglobulin fraction with only minor traces of other plasma proteins, at the same time acceptably influencing the yield, it was chosen as optimal for CCP downstream processing.

**Figure 2:**
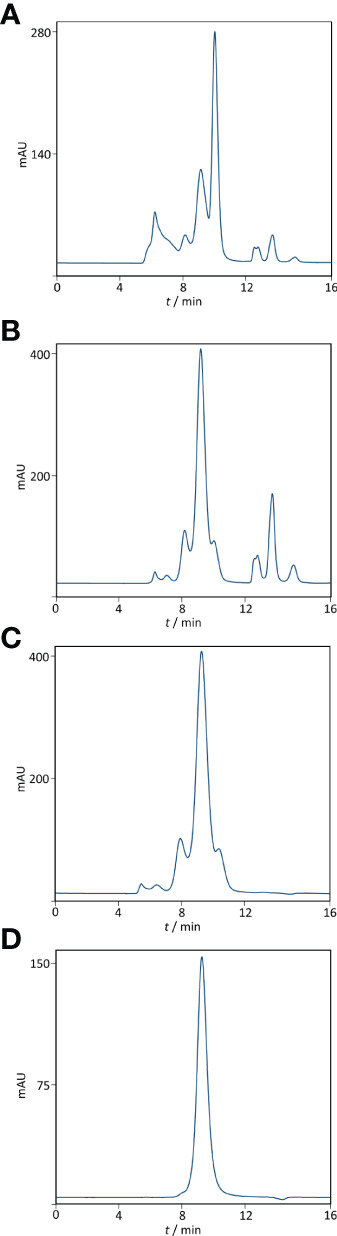
Purity profiling by size-exclusion chromatography. Analysis was performed on TSK-Gel G3000SWXL column (7.8 × 300 mm) with 0.1 M phosphate-sulphate running buffer, pH 6.6, at a flow rate of 1 mL min^-1^. Heat-treated COVID-19 convalescent plasma **(A)**. IgG fraction obtained by caprylic acid precipitation before **(B)** and after diafiltration using a 100 kDa membrane **(C)**. Final product — flow-through fraction from anion-exchange chromatography performed at pH 5.0 (**D**). Detection: UV at 280 nm.

**Figure 3:**
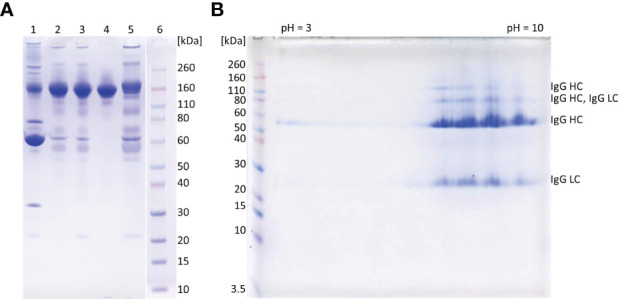
SDS-PAGE analysis of representative samples from purification process. 1D gel electrophoresis **(A)**. Lane 1, COVID-19 convalescent plasma; lane 2, IgG fraction obtained by caprylic acid precipitation; lane 3, IgG fraction after diafiltration; lane 4, flow-through fraction from anion-exchange chromatography (final product); lane 5, elution fraction from anion-exchange chromatography; lane 6, molecular weight standards. The analysis was done on 4-12% Bis-Tris gel under non-reducing conditions. Staining was performed with CBB R250. 2D gel electrophoresis of the final product **(B)**. In the first dimension IgG (*m* = 250 μg) was focused using IPG strip under denaturing conditions (linear pH 3-10). Prior second dimension IPG strip was reduced, alkylated and loaded to a 4-12% gel. Proteins were detected with CBB R250 and identified by MS/MS analysis (LC, light chain; HC, heavy chain). Molecular mass markers are at left side.

Based on ELISA results, 5% caprylic acid enabled preparation of sample with approximately 90% immunoglobulin share in total protein content (**Table 1**). IgG was the most represented isotype (83.2 ± 2.2%), followed by IgA (11.8 ± 3.3%) and IgM (5.2 ± 3.4%). Precipitation had the weakest impact on IgG and IgA, whose yield with respect to the CCP was around 85 and 77%, respectively. On the other hand, it reduced IgM quantity by more than half. SEC analysis of caprylic acid-prepared sample also confirmed substantial depletion of impurities, especially albumin, which was identified according to the retention time, and majority of high molecular weight proteins (**Figures 2A, B**). Its SDS-PAGE profile (**Figure 3A**) corroborated ELISA-determined prevalence of IgG and IgA, exhibiting overlapping electrophoretic mobilities, and traces of IgM, distinguished by much higher molecular weight.

**Table 1:** Share of IgG, IgA and IgM isotypes within total protein or immunoglobulin content in the intermediate fractions and the final product of developed downstream processing protocol, together with their recoveries in every fractionation step.

		IgG	IgA	IgM
**Caprylic acid precipitation**	Share in total proteins [%]	72.5 ± 4.9	9.8 ± 2.6	4.8 ± 3.2
	Share in immunoglobulins [%]	83.2 ± 2.2	11.8 ± 3.3	5.2 ± 3.4
	Recovery in relation to CCP [%]	84.6 ± 6.7	77.4 ± 11.2	42.6 ± 7.4
**Diafiltration**	Share in total proteins [%]	79.2 ± 7.6	10.7 ± 3.0	4.9 ± 3.5
	Share in immunoglobulins [%]	83.6 ± 2.6	12.9 ± 4.0	4.8 ± 3.3
	Recovery in relation to CCP [%]	81.8 ± 8.0	73.8 ± 9.6	40.1 ± 7.5
	Recovery in relation to previous step [%]	96.5 ± 3.2	95.9 ± 4.5	83.0 ± 12.1
**Flow-through chromatography**	Share in total proteins (immunoglobulins) [%]	99.2 ± 0.1	0.7 ± 0.1	0.6 × 10^-3^ ± 0.4 × 10^-3^
	Recovery in relation to CCP [%]	75.1 ± 1.8	4.1 ± 1.5	0.2 ± 0.1
	Recovery in relation to previous step [%]	93.0 ± 7.1	5.6 ± 2.0	1.0 ± 0.8

Samples were submitted to at least three total protein/immunoglobulin concentration measurements for purity and yield calculations. Results are given as a mean from eight independently performed COVID-19 convalescent plasma (CCP) fractionations +/- 95% CI.

Supernatant after precipitation was depleted from the remaining caprylic acid by diafiltration on a 100 kDa membrane. The majority of precipitating agent was removed already after the first diafiltration cycle (**Table 2**). In the final retentate its concentration was in the range or below the method’s limit of quantification (31.3 ± 3.4 ppm). ELISA-determined relative isotype distribution remained the same as in undiafiltrated sample since no considerable loss of either IgG, IgA or IgM during diafiltration occurred (**Table 1**). Immunoglobulin share in total protein content was increased to 95% and IgG purity of almost 80% was achieved due to elimination of small molecular weight proteins/peptides, as evident from SEC analysis (**Figure 2C**).

**Table 2:** Removal of caprylic acid during diafiltration step with indicated recoveries in the filtrates following every cycle and the final preparation (retentate) in relation tostarting, undiafiltrated sample.

	Filtrate 1	Filtrate 2	Filtrate 3	Retentate	Total
**Recovery of caprylic acid in relation to undiafiltrated sample [%]**	84.77 ± 3.10	10.66 ± 1.89	2.46 ± 1.26	0.09 ± 0.05	97.98 ± 4.71

Results are given as a mean from eight experiments +/- 95% CI.

Final polishing step involved anion-exchange chromatography of the sample diafiltrated into the binding buffer (20 mM sodium acetate, pH 5.0). It was performed in the flow-through mode under conditions that allow adsorption of IgA, IgM and other residual impurities exclusively, at the same time enabling passing of IgG as the active principle through the stationary phase without binding. As determined by ELISA, the recovery of the chromatography step was 93.0 ± 7.1% (**Table 1**).

### 3.2 Final Product’s Properties

**Figure 4:**
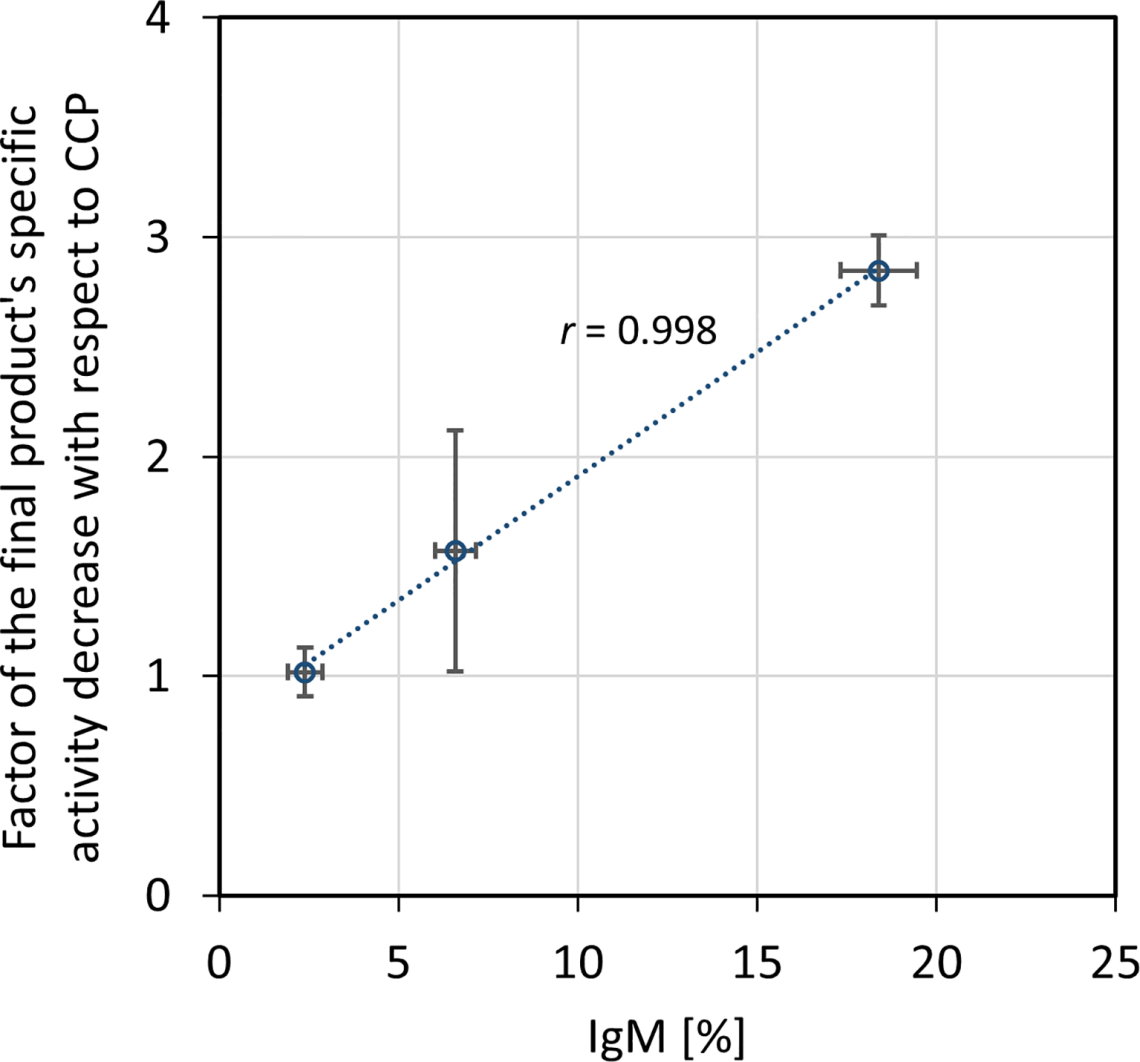
Relationship between factor of the final product’s specific activity decrease in comparison to the COVID-19 convalescent plasma (CCP) and IgM share in the immunoglobulin pool of the starting material. Correlation was assessed by Pearson’s correlation coefficient (*r*) using a significance level of 5%. Specific activity was expressed as neutralization potency, determined by the wild-type SARS-CoV-2 ED_50_ assay, per mg of IgG. The results are given as (geo)mean from eight independently performed refinement procedures ± standard deviation (denoted by error bars).

The ability of CCP or IgG to abolish the virus infectivity as an indirect measure of antibody titer was assessed by the wild-type SARS-CoV-2 ED_50_ assay. IgG yield, determined from neutralization potencies of the CCP and the final product, was 51.0 ± 16.2% (**Table 3**). In comparison to the starting material, the final products exhibited lower specific activities, or neutralization potencies per mg of IgG (**Table 4**). Degree of discrepancy significantly correlated with IgM share in immunoglobulin pool of the respective CCP (*r* = 0.998, *α* = 0.05) (**Figure 4**). Purification factors for every experiment, calculated as a ratio of specific activities, or neutralization potencies per mg of total proteins, of the final product and the starting material, are given in **Table 4**. Purification factor of 3.3-fold was achieved on average (**Table 3**).

**Table 3:** Final product’s properties. Results are given as a mean from eight experiments +/- 95% CI.

SEC-determined purity [%]	SEC-determined aggregate content [%]	ELISA-determined purity [%]	ELISA-determined yield [%]	ED_50_ assay-determined yield [%]	Average purification factor
100	0	99.2 ± 0.1	75.1 ± 1.8	51.0 ± 16.2	3.3

**Table 4:** Comparison of neutralization potencies of the COVID-19 convalescent plasma (CCP A-C) and the corresponding pure IgG preparations determined by the wild-type SARS-CoV-2 ED_50_ assay, together with their specific activities.

	Neutralization potency [IU mL^-1^]	*γ* (IgG) [mg mL^-1^]	*γ* (protein) [mg mL^-1^]	IU mg^-1^ of IgG content	IU mg^-1^ of protein content	Purification fold
**CCP A**	151.8 ± 47.6 (*n* = 5)	9.36	45.25	16.2 ± 5.1	3.4 ± 1.1	
**Pure IgG 1**	176.0 ± 44.8 (*n* = 5)	9.54	9.64	18.4 ± 4.7	18.3 ± 4.7	5.4
**Pure IgG 2**	145.8 ± 38.7 (*n* = 5)	9.70	9.80	15.0 ± 4.0	14.9 ± 4.0	4.4
**Pure IgG 3**	153.0 ± 32.6 (*n* = 3)	10.04	10.14	15.2 ± 3.3	15.1 ± 3.2	4.5
**CCP B**	86.5 ± 13.1 (*n* = 12)	8.64	43.47	10.0 ± 1.5	2.0 ± 0.3	
**Pure IgG 1**	36.9 ± 7.6 (*n* = 6)	10.71	10.82	3.4 ± 0.7	3.4 ± 0.7	1.7
**Pure IgG 2**	32.8 ± 7.8 (*n* = 5)	9.31	9.40	3.5 ± 0.8	3.5 ± 0.8	1.8
**Pure IgG 3**	30.4 ± 6.0 (*n* = 3)	9.52	9.62	3.2 ± 0.6	3.2 ± 0.6	1.6
**CCP C**	575.9 ± 83.2 (*n* = 9)	12.87	67.88	44.7 ± 6.5	8.5 ± 1.2	
**Pure IgG 1**	244.6 ± 21.1 (*n* = 6)	10.60	10.71	23.1 ± 2.0	22.8 ± 2.0	2.7
**Pure IgG 2**	396.8 ± 39.0 (*n* = 5)	10.38	10.49	38.2 ± 3.8	37.8 ± 3.7	4.5

Purification fold is expressed through specific activities of CCP and IgG product from downstream processing exit point, that were calculated as a ratio of neutralization potency and total protein concentration (IU mg^-1^ of protein content). Results are given as a mean from n measurements +/- 95% CI. Specific activity was calculated as a ratio of neutralization potency and IgG (IU mg^-1^ of IgG content) or total protein concentration (IU mg^-1^ of protein content).

The recovery of the whole IgG fraction during overall purification process, determined by ELISA, was 75.1 ± 1.8 (**Tables 1**, **3**). The purity of IgG-based final product was 99.2 ± 0.1%. The remaining IgA was present only in a negligible amount (around 0.7%) (**Table 1**), since its concentration through the procedure was decreased 30.2 ± 8.8-fold. Accordingly, in the SEC analysis, only one peak whose retention time corresponded to the molecular weight of 152.4 ± 1.9 kDa was obtained (**Figure 2D**), matching to that calculated for IgG in CCP (158.6 ± 3.8 kDa) (**Figure 2A**). Furthermore, preparation was completely free from aggregates. The absence of unwanted proteins from the flow-through fraction was confirmed by SDS-PAGE, further proving that IgA and IgM were successfully retained by the column and remained in bound and subsequently eluted fraction (**Figure 3A**). MS/MS analysis of protein spots obtained by 2D gel electrophoresis, intentionally performed by sample overloading for even minor contaminants to appear, provided final proof of the product’s high purity since only peptides from IgG heavy and light chains were detected and identified (**Figure 3B**, **Supplementary Table 1**).

SARS-CoV-2 S protein-specific IgG recovery was measured by ELISA that uses S1 domain as a coating antigen. Every CCP and the corresponding final products were adjusted to the same total IgG concentration, determined as described in Section 2.6.6., and analyzed simultaneously. Their *A*_450 nm_ values were equal, giving the ratio of 1.02 ± 0.01 which indicated that no impoverish or enrichment of the S protein-specific antibodies during refinement procedure occurred.

Subclass distribution in the final product was assessed by ELISA measuring SARS-CoV-2-specific IgG1, IgG2, IgG3 and IgG4 antibodies. Two CCPs and the corresponding final preparations obtained by their independently performed fractionations were adjusted to the same total IgG concentration, determined as described in Section 2.6.6., and analyzed simultaneously. According to the results, expressed as ratio of their *A*_492 nm_ values, processing strategy did not affect relative abundance of any of four IgG subclasses (**Table 5**).

**Table 5:** Final product’s IgG subclass distribution.

	IgG1	IgG2	IgG3	IgG4
**Factor of IgG subclass quantity change in relation to the amount in CCP**	1.10 ± 0.05	1.07 ± 0.11	1.05 ± 0.13	1.04 ± 0.11

Ratio of A_492 nm_ values obtained for the final product and the COVID-19 convalescent plasma (CCP) was used a measure of IgG subclass quantity change. Results are given as mean from four experiments +/- 95% CI. Two plasma units and the corresponding preparations obtained by their independently performed fractionations were adjusted to the same total IgG concentration and analyzed simultaneously by ELISA measuring SARS-CoV-2-specific IgG1, IgG2, IgG3 and IgG4 antibodies.

## Discussion

A highly streamlined, practical and affordable technological platform for the production of immunoglobulin G (IgG) was designed on a small-scale to offer an immediately available treatment option, filling the gap between onset of emerging infectious diseases and development of other specific therapies. Concerning the ongoing pandemic status of COVID-19, the proof of concept was demonstrated on anti-SARS-CoV-2 convalescent plasma, but is likely to be applicable to any other type depending on future needs. Results were recently reported about a single-center, single-blind, placebo-controlled phase I/II clinical trial (NCT04521309) with anti-SARS-CoV-2 IVIGs purified from convalescent plasma in Pakistan, which appeared safe, increased survival, and reduced the disease progression risk (28).

Literature describes numerous modern IgG fractionation methods that generate highly purified and liquid-state stable products of preserved clinical efficacy and are used in practice (13–15, 29). However, they maintain at least some of the deeply rooted cold-ethanol precipitation steps of the Cohn’s method that increase the length of the process, which might pose a problem if greater IgG supply needs to be prepared at short notice. Our production approach is based on a fractionation of fresh frozen plasma sourced from SARS-CoV-2 convalescents. Although robust and employing only caprylic acid precipitation, diafiltration and anion-exchange chromatography, it provided a high-quality product of above the average recovery and without any unwanted proteins or aggregates. Furthermore, the process efficiency has been supported by quantitative data for easy evaluation of the economic feasibility.

The manufacturing procedure was guided by the idea of persistent keeping of IgG as the target molecule in a solution so that protection of its native structure could be assured. Unwanted precipitation of immunoglobulins to a great extent was avoided by employing caprylic acid-mediated fractionation under optimized conditions. It should be borne in mind that caprylic acid-mediated treatment of CCP, although convenient, results in loss of albumin, as well as many other therapeutically useful plasma proteins. But emergent situations when rapid IgG supplies are highly needed could justify its use. Following diafiltration of the supernatant for the removal of the precipitating agent, the purity was boosted to 95% (**Table 1** and **Figure 3A**), which is in the range of that achieved by similar purification protocols (30, 31). But, as a major difference, in our procedure the relative abundance of isotypes in the immunoglobulin-enriched supernatant turned out to be slightly disturbed in comparison to the physiological proportions, mostly because caprylic acid, under employed operating conditions, at least partially succeed to precipitate IgM. It is namely its presence, as well as that of IgA, that causes acute hemolytic (20) or anaphylactic transfusion reactions (19). So, immunoglobulin preparations for intravenous use depleted from IgM and IgA might contribute to the product safety, but their removal poses a challenge for manufacturers as both tend to copurify with IgG during plasma fractionation (32). We have succeeded in their separation by implementing only one anion-exchange step (**Table 1**, **Figures 2**, **3** and **Supplementary Table 1**). So, our chromatographic process could be considered simpler than the one described by Wu et al. (32). For quality insurance of the final preparation 2D gel electrophoresis and MS/MS analysis were performed (**Figure 3B** and **Supplementary Table 1**). It was proved free of IgA and IgM, but also of any other residual impurities, since all protein spots have been attributed to the IgG fragments exclusively.

The developed manufacturing procedure had several advantages worthy of emphasis. According to the ELISA for whole IgG quantification, the overall process efficiency of around 75% (**Tables 1**, **3**) was favorably comparable to some other modern plasma fractionations (13, 15, 30–32), probably due to the reduced number of employed steps. Preservation of SARS-CoV-2 S protein-specific IgG share in the whole IgG population of the final product with respect to the CCP was achieved, as well as relative distribution of all four IgG subclasses (**Table 5**). But, since the availability of convalescent plasma is mostly limited, it would be of value to force the increase of the IgG yield even more, possibly through further optimization of the caprylic acid precipitation that was recognized as the critical point at which the majority of loss occurred, while diafiltration and the final polishing only slightly impaired the recovery.

Unexpectedly, according to the ED_50_ assay, the fractionation approach substantially affected the final product’s capacity to neutralize wild-type SARS-CoV-2 infectivity, reducing it in average by half (**Table 3**), although the manufacturing process conditions did not provoke either redistribution of the specific IgG content or loss of the IgG subclasses (**Table 5**). Similarly, the production of intravenous immunoglobulin used in clinical trial of its safety, tolerability and efficacy for the COVID-19 treatment (https://clinicaltrials.gov/ProvidedDocs/81/NCT04546581/Prot_001.pdf) also resulted in 50% potency loss. Besides, during preparation of anti-SARS-CoV-2 hyperimmune globulin with production steps identical to those for Gamunex^®^, 3-fold decrease in the neutralizing activity occurred (33). We were able to identify that potency loss was due to the removal of IgM from pure IgG preparation. Decrease in potency significantly correlated with the amount of IgM in the respective CCP (**Figure 4**). Further experimental validation through preparation of plasma that would be selectively depleted from the whole IgM fraction is needed. Comparison of its virus-inactivating activity measured pre- and post-depletion should identify biological relevance of the SARS-CoV-2-specific IgMs in the neutralization process and define degree of their functional participation in the overall CCP potency. Contribution of IgM towards the neutralizing capacity of immune serum has been well documented (34–37). It might be even more pronounced than that of IgG due to its pentameric structure and higher valency. Therefore, when it comes to the isotypes other than IgG, all manufacturers should weigh for themselves the benefits against the risks of their co-purification. Loss of neutralization potency due to the removal of IgM might be easily compensated through the preparation of more concentrated final product which is a common practice in current manufacturing of plasma products. Normal intravenous immunoglobulins are commonly presented either as 50 mg mL^-1^ or 100 mg mL^-1^ solutions (38). Accordingly, COVID-19 anti-coronavirus disease hIVIG, in phase 3 of clinical trial, also contains 100 mg mL^-1^ of total proteins (https://clinicaltrials.gov/ProvidedDocs/81/NCT04546581/Prot_001.pdf). Immunoglobulins intended for *i.m.* or *s.c.* use mostly are formulated in concentration as high as 160 mg mL^-1^ (38). Another approach for ensuring sufficient quantity of neutralizing antibodies might include administration of a greater treatment dose.

In conclusion, the fractionation of the CCP was efficiently performed by the sequence of only three steps − precipitation of unwanted proteins, diafiltration of immunoglobulin-enriched fraction and final polishing, resulting in completely pure IgG preparation without IgA, IgM or any other contaminants. During the whole process the active principle was kept in solution which ensured its stability. The overall yield was as good as or even superior to the other yields so far reported. Threefold IgG purification increase from the CCP to the final product was attained. Given that COVID-19 will not be the last pandemic, preparedness plan for the future threats might benefit from small, but easily scalable processes, like the one here described, that would be harmonized with the increased availability of convalescent plasma over a viral outbreak time-course. Namely, it is a well-known fact that ensuring a sustainable supply of safe and high-quality blood and blood products represents a substantial challenge, particularly in times of emerging threats to public health. The need to establish strategies for achieving self-sufficiency has already been recognized (39). Here described process might pose such a mechanism, particularly suitable in epidemic times. Its performance takes just few days in the laboratory setting. Duration of the production cycle might be influenced by scaling-up, but probably to a lesser extent since, from our point of view, fractionation of small batches, collected during shorter period of time and from local community donors, is more advisable in order to assure preparation of the final product boosted with the currently circulating virus variant-specific IgGs. Also, application of pure IgGs, because of their standardized and consistent composition, might make it easier to draw straightforward conclusion coming from carefully designed randomized trials about the benefit of passive immunotherapy, definitive proof of which for the treatment of COVID-19 is still lacking.

## Data Availability Statement

The datasets presented in this study can be found in online repositories. The names of the repository/repositories and accession number(s) can be found below: https://figshare.com/articles/online_resource/LC-MS_MS_data/19430345.

## Ethics Statement

The study involving human participants was reviewed and approved by Croatian Ministry of Health (023-03/20-01/235; permission No. 534-04-3-2/2-20-11). The approval was based on the positive opinion of the Ethical Committee of Croatian Institute of Transfusion Medicine (003-06/20-04/02, opinion No.251-541-06/6-20-2). The patients/participants provided their written informed consent to participate in this study.

## Author Contributions

TK, SR, AŠ, SML and SK performed the experiments. TK, AŠ, SML and SK analyzed the data. TK designed the research and wrote the manuscript. AH organized the convalescent plasma collection. BH was involved in conceptualization, supervision, project administration and funding. All authors contributed to the article and approved the submitted version.

## Funding

The research was funded by the Croatian Science Foundation (grant IP-CORONA-04-2053 to BH), by the European Regional Development Fund, grant number K.K.01.1.1.01.0006, “Strengthening the capacity of CerVirVac for research in virus immunology and vaccinology” and by the European Regional Development Fund − the Competitiveness and Cohesion Operational Programme project, grant number K.K.01.1.1.04.0099, “RAPTOVAX”.

## Conflict of Interest

The authors declare that the research was conducted in the absence of any commercial or financial relationships that could be construed as a potential conflict of interest.

## Publisher’s Note

All claims expressed in this article are solely those of the authors and do not necessarily represent those of their affiliated organizations, or those of the publisher, the editors and the reviewers. Any product that may be evaluated in this article, or claim that may be made by its manufacturer, is not guaranteed or endorsed by the publisher.
