# Supplementary material for: Efficient and Sustainable Platform for Preparation of a High-Quality Immunoglobulin G as an Urgent Treatment Option During Emerging Virus Outbreaks
Source: Front Immunol. 2022 May 17;13:889736. doi: 10.3389/fimmu.2022.889736 (PMC9152316; doi:10.3389/fimmu.2022.889736)
Supplement: Supplementary file 1 [file DataSheet_1.docx]

**S1 Fig. 2D gel electrophoresis of IgG-based final product (same sample as in Fig. 3B) with annotations of protein spots subjected to MS/MS analysis.** List of identified proteins is given in S1 Table.


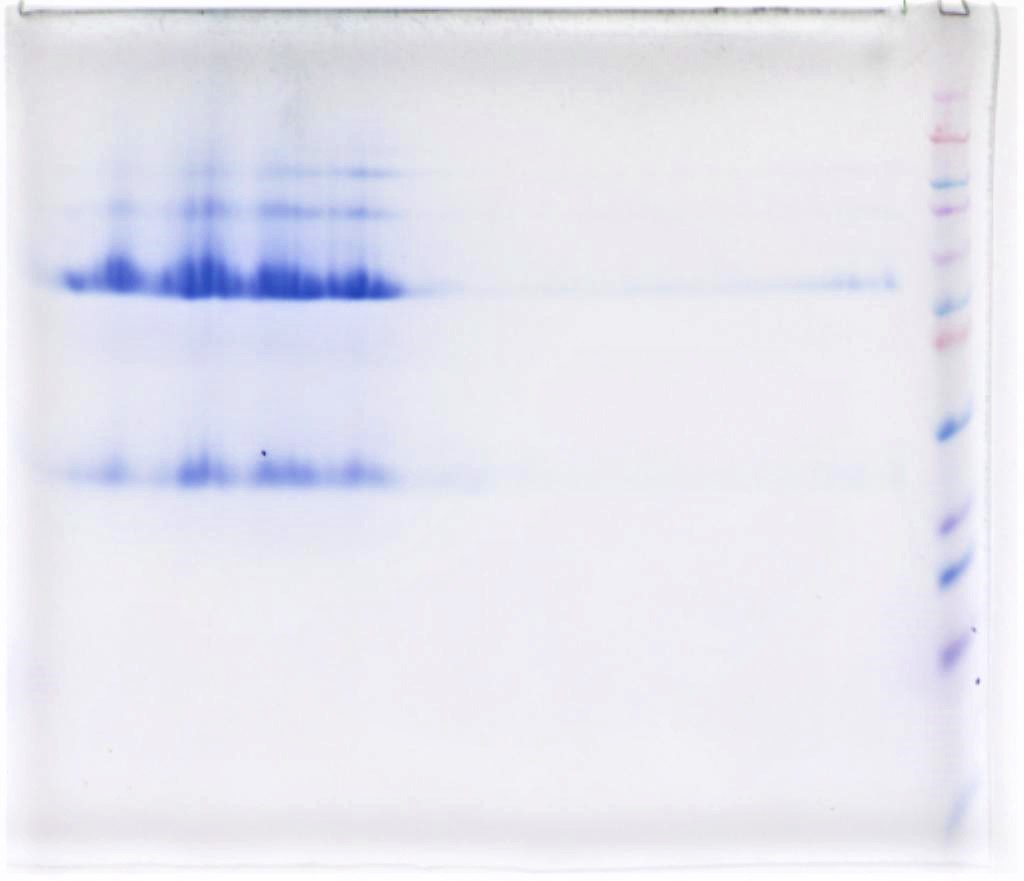


pH = 10

pH = 3

260

[kDa]

80

110

160

6

1

2

3

3.5

10

15

20

30

40

50

60

4

5

11

7

8

9

10

12

**S1 Table. List of proteins identified in the final IgG sample.** Proteins are denoted by numbers as in S1 Fig.

| **Spot** | **Sample**  **(ion[M+H]^+^)** | **Protein name** | **Protein ID** | **Protein score** | **MS/MS score** | **Significance** |  | **Peptide Sequence** |
| --- | --- | --- | --- | --- | --- | --- | --- | --- |
| 1 |  | Immunoglobulin gamma-1 heavy chain | IGG1_HUMAN | 343 |  |  |  |  |
|  | 581.9500 |  |  |  | 81 | yes |  | K.NQVSLTCLVK.G |
|  | 594.2700 |  |  |  | 58 | unique |  | K.GPSVFPLAPSSK.S |
|  | 661.2600 |  |  |  | 107 | yes |  | K.STSGGTAALGCLVK.D |
|  | 839.3500 |  |  |  | 72 | unique |  | K.FNWYVDGVEVHNAK.T |
|  | 904.9500 |  |  |  | 43 | yes |  | R.VVSVLTVLHQDWLNGK.E |
|  | 624.9400 |  |  |  | 15 | unique |  | R.EPQVYTLPPSRDELTK.N |
|  | 625.2600 |  |  |  | 48 | unique |  | K.TTPPVLDSDGSFFLYSK.L |
|  | 713.6100 |  |  |  | 43 | unique |  | R.TPEVTCVVVDVSHEDPEVK.F |
|  | 711.8100 |  |  |  | 26 | unique |  | K.THTCPPCPAPELLGGPSVFLFPPKPK.D |
|  |  | Immunoglobulin heavy constant gamma 3 | IGHG3_HUMAN | 252 |  |  |  |  |
|  | 581.9600 |  |  |  | 86 | yes |  | K.NQVSLTCLVK.G |
|  | 644.2600 |  |  |  | 44 | yes |  | K.GPSVFPLAPCSR.S |
|  | 661.2600 |  |  |  | 107 | yes |  | R.STSGGTAALGCLVK.D |
|  | 904.9500 |  |  |  | 43 | yes |  | R.VVSVLTVLHQDWLNGK.E |
|  | 635.5600 |  |  |  | 15 | yes |  | R.EPQVYTLPPSREEMTK.N |
|  |  | Immunoglobulin heavy constant gamma 4 | IGHG4_HUMAN | 178 |  |  |  |  |
|  | 481.1300 |  |  |  | 8 | unique |  | K.VDKRVESK.Y |
|  | 581.9600 |  |  |  | 86 | yes |  | K.NQVSLTCLVK.G |
|  | 644.2600 |  |  |  | 44 | yes |  | K.GPSVFPLAPCSR.S |
|  | 904.9500 |  |  |  | 43 | yes |  | R.VVSVLTVLHQDWLNGK.E |
|  |  | Immunoglobulin heavy constant gamma 2 | IGHG2_HUMAN | 164 |  |  |  |  |
|  | 581.9500 |  |  |  | 81 | yes |  | K.NQVSLTCLVK.G |
|  | 644.2600 |  |  |  | 44 | yes |  | K.GPSVFPLAPCSR.S |
|  | 712.2800 |  |  |  | 66 | yes |  | R.STSESTAALGCLVK.D |
|  | 897.9900 |  |  |  | 33 | unique |  | R.VVSVLTVVHQDWLNGK.E |
|  | 635.5600 |  |  |  | 15 | yes |  | R.EPQVYTLPPSREEMTK.N |
|  | 635.9000 |  |  |  | 11 | unique |  | K.TTPPMLDSDGSFFLYSK.L |
|  | 759.8100 |  |  |  | 19 | unique |  | R.KCCVECPPCPAPPVAGPSVFLFPPKPK.D |
| 2 |  | Immunoglobulin gamma-1 heavy chain | IGG1_HUMAN | 257 |  |  |  |  |
|  | 581.9700 |  |  |  | 66 | yes |  | K.NQVSLTCLVK.G |
|  | 594.2700 |  |  |  | 42 | unique |  | K.GPSVFPLAPSSK.S |
|  | 643.7700 |  |  |  | 25 | yes |  | R.EPQVYTLPPSR.D |
|  | 661.2800 |  |  |  | 61 | yes |  | K.STSGGTAALGCLVK.D |
|  | 839.3600 |  |  |  | 69 | unique |  | K.FNWYVDGVEVHNAK.T |
|  | 603.9600 |  |  |  | 21 | yes |  | R.VVSVLTVLHQDWLNGK.E |
|  | 624.9300 |  |  |  | 16 | unique |  | R.EPQVYTLPPSRDELTK.N |
|  | 625.2400 |  |  |  | 44 | unique |  | K.TTPPVLDSDGSFFLYSK.L |
|  | 713.6200 |  |  |  | 51 | unique |  | R.TPEVTCVVVDVSHEDPEVK.F |
|  | 712.3200 |  |  |  | 27 | unique |  | K.THTCPPCPAPELLGGPSVFLFPPKPK.D |
|  |  | Immunoglobulin heavy constant gamma 3 | IGHG3_HUMAN | 116 |  |  |  |  |
|  | 581.9700 |  |  |  | 69 | yes |  | K.NQVSLTCLVK.G |
|  | 643.7500 |  |  |  | 20 | yes |  | K.GPSVFPLAPCSR.S |
|  | 643.7700 |  |  |  | 25 | yes |  | R.EPQVYTLPPSR.E |
|  | 644.2600 |  |  |  | 35 | yes |  | K.GPSVFPLAPCSR.S |
|  | 661.2800 |  |  |  | 61 | yes |  | R.STSGGTAALGCLVK.D |
|  | 603.9600 |  |  |  | 21 | yes |  | R.VVSVLTVLHQDWLNGK.E |
|  |  | Immunoglobulin heavy constant gamma 2 | IGHG2_HUMAN | 116 |  |  |  |  |
|  | 581.9700 |  |  |  | 69 | yes |  | K.NQVSLTCLVK.G |
|  | 644.2600 |  |  |  | 35 | yes |  | K.GPSVFPLAPCSR.S |
|  | 643.7700 |  |  |  | 25 | yes |  | R.EPQVYTLPPSR.E |
|  | 712.2700 |  |  |  | 58 | yes |  | R.STSESTAALGCLVK.D |
|  | 599.0500 635.8900 |  |  |  | 13 | unique |  | R.VVSVLTVVHQDWLNGK.E |
|  | 635.8900 |  |  |  | 13 | unique |  | K.TTPPMLDSDGSFFLYSK.L |
|  | 759.8100 |  |  |  | 13 | unique |  | R.KCCVECPPCPAPPVAGPSVFLFPPKPK.D |
| 3 |  | Immunoglobulin gamma-1 heavy chain | IGG1_HUMAN | 288 |  |  |  |  |
|  | 581.9600 |  |  |  | 57 | yes |  | K.NQVSLTCLVK.G |
|  | 594.2800 |  |  |  | 50 | unique |  | K.GPSVFPLAPSSK.S |
|  | 643.7700 |  |  |  | 23 | yes |  | R.EPQVYTLPPSR.D |
|  | 661.2600 |  |  |  | 70 | unique |  | K.STSGGTAALGCLVK.D |
|  | 839.3200 |  |  |  | 67 | unique |  | K.FNWYVDGVEVHNAK.T |
|  | 603.6300 |  |  |  | 42 | yes |  | R.VVSVLTVLHQDWLNGK.E |
|  | 624.9400 |  |  |  | 58 | unique |  | R.EPQVYTLPPSRDELTK.N |
|  | 937.4200 |  |  |  | 82 | unique |  | K.TTPPVLDSDGSFFLYSK.L |
|  | 713.6400 |  |  |  | 51 | unique |  | R.TPEVTCVVVDVSHEDPEVK.F |
|  | 744.0500 |  |  |  | 5 | yes |  | R.VVSVLTVLHQDWLNGKEYK.C |
|  | 711.8200 |  |  |  | 19 | unique |  | K.THTCPPCPAPELLGGPSVFLFPPKPK.D |
| 4 |  | Immunoglobulin gamma-1 heavy chain | IGG1_HUMAN | 393 |  |  |  |  |
|  | 581.9500 |  |  |  | 65 | yes |  | K.NQVSLTCLVK.G |
|  | 594.2600 |  |  |  | 58 | unique |  | K.GPSVFPLAPSSK.S |
|  | 643.7700 |  |  |  | 10 | yes |  | R.EPQVYTLPPSR.D |
|  | 661.2600 |  |  |  | 78 | yes |  | K.STSGGTAALGCLVK.D |
|  | 839.3200 |  |  |  | 78 | unique |  | K.FNWYVDGVEVHNAK.T |
|  | 904.4500 |  |  |  | 80 | yes |  | R.VVSVLTVLHQDWLNGK.E |
|  | 624.9300 |  |  |  | 17 | unique |  | R.EPQVYTLPPSRDELTK.N |
|  | 625.2500 |  |  |  | 59 | unique |  | K.TTPPVLDSDGSFFLYSK.L |
|  | 713.6200 |  |  |  | 55 | unique |  | R.TPEVTCVVVDVSHEDPEVK.F |
|  | 949.4500 |  |  |  | 29 | unique |  | K.THTCPPCPAPELLGGPSVFLFPPKPK.D |
|  | 667.6700 |  |  |  | 8 | unique |  | K.SCDKTHTCPPCPAPELLGGPSVFLFPPKPK.D |
|  |  | Immunoglobulin lambda constant 2 | IGLC2_HUMAN | 191 |  |  |  |  |
|  | 872.3600 |  |  |  | 54 | unique |  | K.YAASSYLSLTPEQWK.S |
|  | 662.6100 |  |  |  | 13 | unique |  | K.AAPSVTLFPPSSEELQANK.A |
|  | 1106.0000 |  |  |  | 103 | unique |  | K.ATLVCLISDFYPGAVTVAWK.A |
|  |  | Immunoglobulin heavy constant gamma 3 | IGHG3_HUMAN | 169 |  |  |  |  |
|  | 581.9500 |  |  |  | 65 | yes |  | K.NQVSLTCLVK.G |
|  | 643.7600 |  |  |  | 20 | yes |  | R.EPQVYTLPPSR.E |
|  | 644.2700 |  |  |  | 38 | yes |  | K.GPSVFPLAPCSR.S |
|  | 661.2600 |  |  |  | 78 | yes |  | R.STSGGTAALGCLVK.D |
|  | 904.4500 |  |  |  | 80 | yes |  | R.VVSVLTVLHQDWLNGK.E |
|  | 635.5700 |  |  |  | 14 | yes |  | R.EPQVYTLPPSREEMTK.N |
|  |  | Immunoglobulin heavy constant gamma 4 | IGHG4_HUMAN | 151 |  |  |  |  |
|  | 581.9500 |  |  |  | 65 | yes |  | K.NQVSLTCLVK.G |
|  | 644.2700 |  |  |  | 38 | yes |  | K.GPSVFPLAPCSR.S |
|  | 712.2600 |  |  |  | 60 | yes |  | R.STSESTAALGCLVK.D |
|  | 904.4500 |  |  |  | 80 | yes |  | R.VVSVLTVLHQDWLNGK.E |
|  | 701.2400 |  |  |  | 4 | unique |  | R.WQEGNVFSCSVMHEALHNHYTQK.S |
|  |  | Immunoglobulin kappa constant | IGKC_HUMAN | 143 |  |  |  |  |
|  | 752.3100 |  |  |  | 63 | unique |  | K.DSTYSLSSTLTLSK.A |
|  | 899.9100 |  |  |  | 51 | unique |  | K.SGTASVVCLLNNFYPR.E |
|  | 625.9200 |  |  |  | 52 | unique |  | K.VYACEVTHQGLSSPVTK.S |
|  | 649.2600 |  |  |  | 32 | unique |  | R.TVAAPSVFIFPPSDEQLK.S |
|  |  | Immunoglobulin heavy constant gamma 2 | IGHG2_HUMAN | 110 |  |  |  |  |
|  | 581.9500 |  |  |  | 71 | yes |  | K.NQVSLTCLVK.G |
|  | 643.7600 |  |  |  | 20 | yes |  | R.EPQVYTLPPSR.E |
|  | 644.2700 |  |  |  | 38 | yes |  | K.GPSVFPLAPCSR.S |
|  | 712.2600 |  |  |  | 60 | yes |  | R.STSESTAALGCLVK.D |
|  | 635.5700 |  |  |  | 14 | yes |  | R.EPQVYTLPPSREEMTK.N |
|  | 759.8200 |  |  |  | 18 | unique |  | R.KCCVECPPCPAPPVAGPSVFLFPPKPK.D |
| 5 |  | Immunoglobulin gamma-1 heavy chain | IGG1_HUMAN | 394 |  |  |  |  |
|  | 581.9600 |  |  |  | 71 | yes |  | K.NQVSLTCLVK.G |
|  | 594.2700 |  |  |  | 81 | unique |  | K.GPSVFPLAPSSK.S |
|  | 643.7700 |  |  |  | 28 | yes |  | R.EPQVYTLPPSR.D |
|  | 661.2600 |  |  |  | 89 | yes |  | K.STSGGTAALGCLVK.D |
|  | 839.3600 |  |  |  | 67 | unique |  | K.FNWYVDGVEVHNAK.T |
|  | 904.4900 |  |  |  | 58 | yes |  | R.VVSVLTVLHQDWLNGK.E |
|  | 624.9400 |  |  |  | 14 | unique |  | R.EPQVYTLPPSRDELTK.N |
|  | 937.4100 |  |  |  | 54 | unique |  | K.TTPPVLDSDGSFFLYSK.L |
|  | 713.6100 |  |  |  | 55 | unique |  | R.TPEVTCVVVDVSHEDPEVK.F |
|  | 744.0000 |  |  |  | 16 | yes |  | R.VVSVLTVLHQDWLNGKEYK.C |
|  | 949.4700 |  |  |  | 41 | unique |  | K.THTCPPCPAPELLGGPSVFLFPPKPK.D |
|  |  | Immunoglobulin heavy constant gamma 3 | IGHG3_HUMAN | 151 |  |  |  |  |
|  | 581.9800 |  |  |  | 55 | yes |  | K.NQVSLTCLVK.G |
|  | 643.7700 |  |  |  | 28 | yes |  | R.EPQVYTLPPSR.E |
|  | 644.2700 |  |  |  | 19 | yes |  | K.GPSVFPLAPCSR.S |
|  | 661.2600 |  |  |  | 89 | yes |  | R.STSGGTAALGCLVK.D |
|  | 904.4900 |  |  |  | 58 | yes |  | R.VVSVLTVLHQDWLNGK.E |
|  | 635.9100 |  |  |  | 17 | yes |  | R.EPQVYTLPPSREEMTK.N |
|  | 744.0000 |  |  |  | 16 | yes |  | R.VVSVLTVLHQDWLNGKEYK.C |
| 6 |  | Immunoglobulin gamma-1 heavy chain | IGG1_HUMAN | 360 |  |  |  |  |
|  | 581.9600 |  |  |  | 71 | yes |  | K.NQVSLTCLVK.G |
|  | 594.2700 |  |  |  | 66 | unique |  | K.GPSVFPLAPSSK.S |
|  | 661.2900 |  |  |  | 77 | yes |  | K.STSGGTAALGCLVK.D |
|  | 839.3600 |  |  |  | 68 | unique |  | K.FNWYVDGVEVHNAK.T |
|  | 904.9700 |  |  |  | 70 | yes |  | R.VVSVLTVLHQDWLNGK.E |
|  | 624.9400 |  |  |  | 13 | unique |  | R.EPQVYTLPPSRDELTK.N |
|  | 625.2700 |  |  |  | 67 | unique |  | K.TTPPVLDSDGSFFLYSK.L |
|  | 625.2900 |  |  |  | 17 | unique |  | R.EPQVYTLPPSRDELTK.N |
|  | 713.6400 |  |  |  | 57 | unique |  | R.TPEVTCVVVDVSHEDPEVK.F |
|  | 948.7500 |  |  |  | 37 | unique |  | K.THTCPPCPAPELLGGPSVFLFPPKPK.D |
|  | 834.3800 |  |  |  | 17 | unique |  | K.SCDKTHTCPPCPAPELLGGPSVFLFPPKPK.D |
|  |  | Immunoglobulin heavy constant gamma 3 | IGHG3_HUMAN | 174 |  |  |  |  |
|  | 581.9700 |  |  |  | 71 | yes |  | K.NQVSLTCLVK.G |
|  | 661.2900 |  |  |  | 77 | yes |  | R.STSGGTAALGCLVK.D |
|  | 904.9700 |  |  |  | 70 | yes |  | R.VVSVLTVLHQDWLNGK.E |
|  | 635.5900 |  |  |  | 21 | unique |  | R.EPQVYTLPPSREEMTK.N |
| 7 |  | Immunoglobulin gamma-1 heavy chain | IGG1_HUMAN | 486 |  |  |  |  |
|  | 581.9600 |  |  |  | 61 | yes |  | K.NQVSLTCLVK.G |
|  | 594.2600 |  |  |  | 45 | unique |  | K.GPSVFPLAPSSK.S |
|  | 643.7700 |  |  |  | 21 | yes |  | R.EPQVYTLPPSR.D |
|  | 661.2700 |  |  |  | 98 | yes |  | K.STSGGTAALGCLVK.D |
|  | 839.3600 |  |  |  | 72 | unique |  | K.FNWYVDGVEVHNAK.T |
|  | 904.9400 |  |  |  | 49 | yes |  | R.VVSVLTVLHQDWLNGK.E |
|  | 624.9400 |  |  |  | 20 | unique |  | R.EPQVYTLPPSRDELTK.N |
|  | 937.4000 |  |  |  | 96 | unique |  | K.TTPPVLDSDGSFFLYSK.L |
|  | 713.6200 |  |  |  | 47 | unique |  | R.TPEVTCVVVDVSHEDPEVK.F |
|  | 948.7500 |  |  |  | 47 | unique |  | K.THTCPPCPAPELLGGPSVFLFPPKPK.D |
|  | 834.6000 |  |  |  | 25 | unique |  | K.SCDKTHTCPPCPAPELLGGPSVFLFPPKPK.D |
|  | 950.6100 |  |  |  | 5 | unique |  | R.TPEVTCVVVDVSHEDPEVKFNWYVDGVEVHNAK.T |
|  |  | Immunoglobulin heavy constant gamma 3 | IGHG3_HUMAN | 221 |  |  |  |  |
|  | 581.9600 |  |  |  | 61 | yes |  | K.NQVSLTCLVK.G |
|  | 643.7600 |  |  |  | 19 | yes |  | R.EPQVYTLPPSR.E |
|  | 644.2700 |  |  |  | 51 | yes |  | K.GPSVFPLAPCSR.S |
|  | 661.2700 |  |  |  | 98 | yes |  | R.STSGGTAALGCLVK.D |
|  | 0 904.9400 |  |  |  | 49 | yes |  | R.VVSVLTVLHQDWLNGK.E |
|  | 635.5900 |  |  |  | 31 | yes |  | R.EPQVYTLPPSREEMTK.N |
|  | 718.0400 |  |  |  | 9 | unique |  | R.CPAPELLGGPSVFLFPPKPK.D |
|  |  | Immunoglobulin heavy constant gamma 2 | IGHG2_HUMAN | 160 |  |  |  |  |
|  | 581.9500 |  |  |  | 68 | yes |  | K.NQVSLTCLVK.G |
|  | 643.7600 |  |  |  | 19 | yes |  | R.EPQVYTLPPSR.E |
|  | 644.2700 |  |  |  | 51 | yes |  | K.GPSVFPLAPCSR.S |
|  | 712.2900 |  |  |  | 60 | yes |  | R.STSESTAALGCLVK.D |
|  | 897.9400 |  |  |  | 53 | unique |  | R.VVSVLTVVHQDWLNGK.E |
|  | 635.5900 |  |  |  | 31 | yes |  | R.EPQVYTLPPSREEMTK.N |
|  | 953.3800 |  |  |  | 42 | unique |  | K.TTPPMLDSDGSFFLYSK.L |
|  | 727.7700 |  |  |  | 5 | unique |  | K.CCVECPPCPAPPVAGPSVFLFPPKPK.D |
|  | 759.8200 |  |  |  | 10 | unique |  | R.KCCVECPPCPAPPVAGPSVFLFPPKPK.D |
| 8 |  | Immunoglobulin gamma-1 heavy chain | IGG1_HUMAN | 569 |  |  |  |  |
|  | 581.9700 |  |  |  | 69 | yes |  | Peptide  381 581.9700 1161.9254 1160.6223 1.3031 0 (69) 0.00051 1 K.NQVSLTCLVK.G |
|  | 594.2800 |  |  |  | 49 | unique |  | K.GPSVFPLAPSSK.S |
|  | 643.7800 |  |  |  | 30 | yes |  | R.EPQVYTLPPSR.D |
|  | 661.2700 |  |  |  | 116 | yes |  | K.STSGGTAALGCLVK.D |
|  | 839.3800 |  |  |  | 82 | unique |  | K.FNWYVDGVEVHNAK.T |
|  | 904.9600 |  |  |  | 88 | yes |  | R.VVSVLTVLHQDWLNGK.E |
|  | 624.9300 |  |  |  | 22 | unique |  | R.EPQVYTLPPSRDELTK.N |
|  | 937.4200 |  |  |  | 83 | unique |  | K.TTPPVLDSDGSFFLYSK.L |
|  | 713.6400 |  |  |  | 43 | unique |  | R.TPEVTCVVVDVSHEDPEVK.F |
|  | 743.2900 |  |  |  | 14 | yes |  | R.VVSVLTVLHQDWLNGKEYK.C |
|  | 701.0100 |  |  |  | 10 | yes |  | R.WQQGNVFSCSVMHEALHNHYTQK.S |
|  | 948.4700 |  |  |  | 34 | unique |  | K.THTCPPCPAPELLGGPSVFLFPPKPK.D |
|  | 834.8600 |  |  |  | 50 | unique |  | K.SCDKTHTCPPCPAPELLGGPSVFLFPPKPK.D |
|  | 950.7000 |  |  |  | 23 | unique |  | R.TPEVTCVVVDVSHEDPEVKFNWYVDGVEVHNAK.T |
|  |  | Immunoglobulin heavy constant gamma 3 | IGHG3_HUMAN | 322 |  |  |  |  |
|  | 581.9800 |  |  |  | 91 | yes |  | K.NQVSLTCLVK.G |
|  | 643.7800 |  |  |  | 30 | yes |  | R.EPQVYTLPPSR.E |
|  | 644.2500 |  |  |  | 46 | yes |  | K.GPSVFPLAPCSR.S |
|  | 661.2700 |  |  |  | 116 | yes |  | R.STSGGTAALGCLVK.D |
|  | 904.9600 |  |  |  | 88 | yes |  | R.VVSVLTVLHQDWLNGK.E |
|  | 635.5700 |  |  |  | 18 | yes |  | R.EPQVYTLPPSREEMTK.N |
|  |  | Immunoglobulin heavy constant gamma 4 | IGHG4_HUMAN | 230 |  |  |  |  |
|  | 581.9800 |  |  |  | 91 | yes |  | K.NQVSLTCLVK.G |
|  | 644.2500 |  |  |  | 46 | yes |  | K.GPSVFPLAPCSR.S |
|  | 712.2900 |  |  |  | 63 | yes |  | R.STSESTAALGCLVK.D |
|  | 904.9600 |  |  |  | 88 | yes |  | R.VVSVLTVLHQDWLNGK.E |
|  | 743.2900 |  |  |  | 14 | yes |  | R.VVSVLTVLHQDWLNGKEYK.C |
|  |  | Immunoglobulin heavy constant gamma 2 | IGHG2_HUMAN | 200 |  |  |  |  |
|  | 581.9800 1 |  |  |  | 91 | yes |  | K.NQVSLTCLVK.G |
|  | 643.7800 |  |  |  | 30 | yes |  | R.EPQVYTLPPSR.E |
|  | 644.2500 |  |  |  | 46 | yes |  | K.GPSVFPLAPCSR.S |
|  | 712.2900 |  |  |  | 63 | yes |  | R.STSESTAALGCLVK.D |
|  | 599.0200 |  |  |  | 40 | unique |  | R.VVSVLTVVHQDWLNGK.E |
|  | 635.5700 |  |  |  | 18 | yes |  | R.EPQVYTLPPSREEMTK.N |
|  | 635.9300 |  |  |  | 44 | unique |  | K.TTPPMLDSDGSFFLYSK.L |
|  | 701.0100 |  |  |  | 10 | yes |  | R.WQQGNVFSCSVMHEALHNHYTQK.S |
|  | 970.8000 |  |  |  | 43 | unique |  | K.CCVECPPCPAPPVAGPSVFLFPPKPK.D |
|  | 760.3200 |  |  |  | 16 | unique |  | R.KCCVECPPCPAPPVAGPSVFLFPPKPK.D |
|  | 950.7000 |  |  |  | 23 | unique |  | R.TPEVTCVVVDVSHEDPEVQFNWYVDGVEVHNAK.T |
|  |  | Immunoglobulin heavy variable 3-30-5 | HVC05_HUMAN | 111 |  |  |  |  |
|  | 645.7500 |  |  |  | 71 | unique |  | R.AEDTAVYYCAK.- |
|  | 676.8000 |  |  |  | 69 | unique |  | K.NTLYLQMNSLR.A |
| 9 |  | Immunoglobulin gamma-1 heavy chain | IGG1_HUMAN | 457 |  |  |  |  |
|  | 581.9700 |  |  |  | 71 | yes |  | K.NQVSLTCLVK.G |
|  | 594.2800 |  |  |  | 71 | unique |  | K.GPSVFPLAPSSK.S |
|  | 643.7800 |  |  |  | 17 | yes |  | R.EPQVYTLPPSR.D |
|  | 661.2700 |  |  |  | 97 | yes |  | K.STSGGTAALGCLVK.D |
|  | 839.3600 |  |  |  | 74 | unique |  | K.FNWYVDGVEVHNAK.T |
|  | 905.4800 |  |  |  | 45 | yes |  | R.VVSVLTVLHQDWLNGK.E |
|  | 624.9600 |  |  |  | 17 | unique |  | R.EPQVYTLPPSRDELTK.N |
|  | 937.4100 |  |  |  | 60 | unique |  | K.TTPPVLDSDGSFFLYSK.L |
|  | 713.6300 |  |  |  | 42 | unique |  | R.TPEVTCVVVDVSHEDPEVK.F |
|  | 849.0900 |  |  |  | 9 | yes |  | K.GFYPSDIAVEWESNGQPENNYK.T |
|  | 711.8300 |  |  |  | 36 | unique |  | K.THTCPPCPAPELLGGPSVFLFPPKPK.D |
|  | 834.3700 |  |  |  | 11 | unique |  | K.SCDKTHTCPPCPAPELLGGPSVFLFPPKPK.D |
|  | 950.6900 |  |  |  | 4 | unique |  | R.TPEVTCVVVDVSHEDPEVKFNWYVDGVEVHNAK.T |
|  |  | Immunoglobulin heavy constant gamma 3 | IGHG3_HUMAN | 215 |  |  |  |  |
|  | 581.9700 |  |  |  | 70 | yes |  | K.NQVSLTCLVK.G |
|  | 594.1500 |  |  |  | 12 | unique |  | K.SCDTPPPCPR.C |
|  | 643.7800 |  |  |  | 17 | yes |  | R.EPQVYTLPPSR.E |
|  | 644.2800 |  |  |  | 40 | yes |  | K.GPSVFPLAPCSR.S |
|  | 661.2700 |  |  |  | 97 | yes |  | R.STSGGTAALGCLVK.D |
|  | 603.6600 |  |  |  | 45 | yes |  | R.VVSVLTVLHQDWLNGK.E |
|  | 635.2800 |  |  |  | 21 | yes |  | R.EPQVYTLPPSREEMTK.N |
|  |  | Immunoglobulin heavy constant gamma 4 | IGHG4_HUMAN | 132 |  |  |  |  |
|  | 581.9700 |  |  |  | 70 | yes |  | K.NQVSLTCLVK.G |
|  | 644.2800 |  |  |  | 40 | yes |  | K.GPSVFPLAPCSR.S |
|  | 712.2900 |  |  |  | 42 | yes |  | R.STSESTAALGCLVK.D |
|  | 905.4800 |  |  |  | 45 | yes |  | R.VVSVLTVLHQDWLNGK.E |
| 10 |  | Immunoglobulin kappa constant | IGKC_HUMAN | 576 |  |  |  |  |
|  | 435.8600 |  |  |  | 7 | yes |  | K.SFNRGEC.- |
|  | 751.8400 |  |  |  | 71 | yes |  | K.DSTYSLSSTLTLSK.A |
|  | 600.3200 |  |  |  | 51 | yes |  | K.SGTASVVCLLNNFYPR.E |
|  | 938.3800 |  |  |  | 68 | yes |  | K.VYACEVTHQGLSSPVTK.S |
|  | 973.4700 |  |  |  | 59 | unique |  | R.TVAAPSVFIFPPSDEQLK.S |
|  | 712.6000 |  |  |  | 55 | yes |  | K.VDNALQSGNSQESVTEQDSK.D |
|  | 714.2900 |  |  |  | 35 | yes |  | K.HKVYACEVTHQGLSSPVTK.S |
|  |  | Immunoglobulin kappa light chain | IGK_HUMAN | 488 |  |  |  |  |
|  | 435.8600 |  |  |  | 7 | yes |  | K.SFNRGEC.- |
|  | 751.8400 |  |  |  | 71 | yes |  | K.DSTYSLSSTLTLSK.A |
|  | 899.9300 |  |  |  | 53 | yes |  | K.SGTASVVCLLNNFYPR.E |
|  | 938.3800 |  |  |  | 68 | yes |  | K.VYACEVTHQGLSSPVTK.S |
|  | 668.2900 |  |  |  | 17 | unique |  | K.GTVAAPSVFIFPPSDEQLK.S |
|  | 713.2500 |  |  |  | 75 | yes |  | K.VDNALQSGNSQESVTEQDSK.D |
|  | 714.2900 |  |  |  | 35 | yes |  | K.HKVYACEVTHQGLSSPVTK.S |
|  |  | Immunoglobulin lambda constant 2 | IGLC2_HUMAN | 369 |  |  |  |  |
|  | 864.3800 |  |  |  | 18 | unique |  | K.TVAPTECS.- |
|  | 856.3600 |  |  |  | 69 | unique |  | R.SYSCQVTHEGSTVEK.T |
|  | 582.6000 |  |  |  | 36 | unique |  | K.YAASSYLSLTPEQWK.S |
|  | 993.4400 |  |  |  | 52 | unique |  | K.AAPSVTLFPPSSEELQANK.A |
|  | 1106.5600 |  |  |  | 113 | unique |  | K.ATLVCLISDFYPGAVTVAWK.A |
|  |  | Immunoglobulin kappa variable 3D-20 | KVD20_HUMAN | 134 |  |  |  |  |
|  | 816.8500 |  |  |  | 101 | unique |  | R.FSGSGSGTDFTLTISR.L |
| 11 |  | Immunoglobulin kappa constant | IGKC_HUMAN | 393 |  |  |  |  |
|  | 751.8300 |  |  |  | 72 | unique |  | K.DSTYSLSSTLTLSK.A |
|  | 600.3300 |  |  |  | 53 | unique |  | K.SGTASVVCLLNNFYPR.E |
|  | 938.3600 |  |  |  | 79 | unique |  | K.VYACEVTHQGLSSPVTK.S |
|  | 973.4600 |  |  |  | 48 | unique |  | R.TVAAPSVFIFPPSDEQLK.S |
|  | 712.5800 |  |  |  | 56 | unique |  | K.VDNALQSGNSQESVTEQDSK.D |
|  | 906.1400 |  |  |  | 12 | unique |  | K.VDNALQSGNSQESVTEQDSKDSTYSLSSTLTLSK.A |
|  |  | Immunoglobulin lambda constant 2 | IGLC2_HUMAN | 232 |  |  |  |  |
|  | 872.3700 |  |  |  | 46 | unique |  | K.YAASSYLSLTPEQWK.S |
|  | 662.6100 |  |  |  | 25 | unique |  | K.AAPSVTLFPPSSEELQANK.A |
|  | 1106.0200 |  |  |  | 85 | unique |  | K.ATLVCLISDFYPGAVTVAWK.A |
|  |  | Immunoglobulin kappa variable 3D-20 | KVD20_HUMAN | 142 |  |  |  |  |
|  | 519.9300 |  |  |  | 42 | unique |  | R.LLIYDASSR.A |
|  | 816.8200 |  |  |  | 92 | yes |  | R.FSGSGSGTDFTLTISR.L |
|  | 816.8200 |  | KV320_HUMAN | 134 | 92 | yes |  | R.FSGSGSGTDFTLTISR.L |
|  | 814.0100 |  |  |  | 33 | unique |  | R.ASQSVSSSYLAWYQQKPGQAPR.L |
| 12 |  | Immunoglobulin kappa constant | IGKC_HUMAN | 456 |  |  |  |  |
|  | 435.8300 |  |  |  | 21 | yes |  | K.SFNRGEC.- |
|  | 751.8400 |  |  |  | 80 | yes |  | K.DSTYSLSSTLTLSK.A |
|  | 600.6700 |  |  |  | 61 | yes |  | K.SGTASVVCLLNNFYPR.E |
|  | 938.4300 |  |  |  | 73 | yes |  | K.VYACEVTHQGLSSPVTK.S |
|  | 973.4400 |  |  |  | 39 | unique |  | R.TVAAPSVFIFPPSDEQLK.S |
|  | 701.3000 |  |  |  | 23 | unique |  | -.RTVAAPSVFIFPPSDEQLK.S |
|  | 712.5900 |  |  |  | 68 | yes |  | K.VDNALQSGNSQESVTEQDSK.D |
|  | 714.6500 |  |  |  | 46 | yes |  | K.HKVYACEVTHQGLSSPVTK.S |
|  | 905.9000 |  |  |  | 27 | yes |  | K.VDNALQSGNSQESVTEQDSKDSTYSLSSTLTLSK.A |
|  |  | Immunoglobulin kappa light chain | IGK_HUMAN | 424 |  |  |  |  |
|  | 435.8300 |  |  |  | 21 | yes |  | K.SFNRGEC.- |
|  | 751.8400 |  |  |  | 80 | yes |  | K.DSTYSLSSTLTLSK.A |
|  | 600.6700 |  |  |  | 61 | yes |  | K.SGTASVVCLLNNFYPR.E |
|  | 938.4300 |  |  |  | 73 | yes |  | K.VYACEVTHQGLSSPVTK.S |
|  | 668.3000 |  |  |  | 25 | unique |  | K.GTVAAPSVFIFPPSDEQLK.S |
|  | 712.5900 |  |  |  | 68 | yes |  | K.VDNALQSGNSQESVTEQDSK.D |
|  | 714.6500 |  |  |  | 46 | yes |  | K.HKVYACEVTHQGLSSPVTK.S |
|  | 905.9000 |  |  |  | 27 | yes |  | K.VDNALQSGNSQESVTEQDSKDSTYSLSSTLTLSK.A |
|  |  | Immunoglobulin lambda constant 2 | IGLC2_HUMAN | 195 |  |  |  |  |
|  | 872.3900 |  |  |  | 69 | unique |  | K.YAASSYLSLTPEQWK.S |
|  | 662.6200 |  |  |  | 23 | unique |  | K.AAPSVTLFPPSSEELQANK.A |
|  | 1106.0400 |  |  |  | 102 | unique |  | K.ATLVCLISDFYPGAVTVAWK.A |
|  |  | Immunoglobulin kappa variable 3D-20 | KVD20_HUMAN | 114 |  |  |  |  |
|  | 816.8500 |  |  |  | 85 | unique |  | R.FSGSGSGTDFTLTISR.L |
